# Transparency, Accessibility, and Variability of US Hospital Price Data

**DOI:** 10.1001/jamanetworkopen.2021.10109

**Published:** 2021-05-14

**Authors:** Waqas Haque, Muzzammil Ahmadzada, Hassan Allahrakha, Eman Haque, David Hsiehchen

**Affiliations:** 1 University of Texas Southwestern Medical School, Dallas; 2 Johns Hopkins University, Baltimore, Maryland; 3 William Carey College of Osteopathic Medicine, Hattiesburg, Mississippi; 4 Southern Methodist University, Dallas, Texas; 5 Division of Hematology and Oncology, Department of Internal Medicine, University of Texas Southwestern Medical Center, Dallas

## Abstract

This cross-sectional study evaluates the compliance of hospitals with a Centers for Medicare & Medicaid Services ruling mandating that hospital chargemasters be publicly available in a machine-readable file.

## Introduction

The United States spends a greater proportion of its gross domestic product on health care than any other nation, which is partly due to the excessive price of goods and services.^1^ Price transparency may empower consumers to seek lower-cost care and lead high-cost health care professionals and systems to decrease prices.^2,3,4^ The Centers for Medicare & Medicaid Services (CMS) ruling CMS-1694-F, effective on January 1, 2019, mandated that hospital chargemasters be publicly available in a machine-readable file. Herein, we assessed the compliance of hospitals with this CMS ruling 18 months after its effective date.

## Methods

This cross-sectional study followed the Strengthening the Reporting of Observational Studies in Epidemiology (STROBE) reporting guideline. Per the Common Rule, institutional review board approval was not sought because this study is not human participant research. Hospital characteristics were obtained from the CMS website. US hospitals were eligible for inclusion if an institution website was identified. For chargemasters that could not be identified from the institution website, a Google search was performed using the name of the hospital and the terms *chargemaster*, *charge description master*, *charge list*, *price list*, *standard price*, or *standard charges*. To assess the accuracy of a single reviewer assessing chargemaster prevalence, 200 hospitals were reviewed by 2 reviewers (M.A. and E.H.) independently, with 92% agreement (Cohen κ = 0.84), indicating very high concordance. Two undergraduates served as proxies for layperson internet users and reviewed 25 chargemaster items, including common medications and mandatory shoppable services, as defined by CMS.^5^ Data abstraction was performed between June 30 and November 20, 2020. Logistic regression analysis was used to determine factors associated with hospital compliance to the CMS ruling. Statistical significance was set at *P* < .05, and all hypothesis tests were 2-tailed. All statistical analyses were performed using Stata version 16 (StataCorp).

## Results

Among the 5288 US hospitals associated with a website, most were located in urban settings (2954 [55.9%]), had fewer than 100 beds (2701 [51.1%]), and were classified as acute care facilities (3255 [61.6%]) (Table). A total of 2723 hospitals (51.5%) did not have an online chargemaster in a machine-readable format, including 305 hospitals (5.8%) with broken links or incorrectly linked files and 138 hospitals (2.6%) that only provided an online cost estimator. The median (interquartile range) number of clicks needed to reach the chargemaster from the institution website homepage was 3 (2-8).

**Table.** Factors Associated With Hospital Compliance to the Centers for Medicare & Medicaid Services Price Transparency Rule

Hospital characteristic	No. (%)	Univariable analysis		Multivariable analysis	
		OR (95% CI)	*P* value	OR (95% CI)	*P* value
Urban vs rural					
Rural	2334 (44.1)	1 [Reference]	NA	1 [Reference]	NA
Urban	2954 (55.9)	1.01 (0.90-1.12)	.90	1.12 (0.95-1.31)	.17
Hospital size					
Small, <100 beds	2701 (51.1)	1 [Reference]	NA	1 [Reference]	NA
Medium, 100-300 beds	1544 (29.2)	1.26 (1.11-1.44)	<.001	1.06 (0.90-1.26)	.49
Large, >300 beds	1043 (19.7)	1.44 (1.23-1.68)	<.001	1.10 (0.90-1.35)	.34
Emergency services capable					
No	847 (16.0)	1 [Reference]	NA	1 [Reference]	NA
Yes	4440 (84.0)	2.38 (2.04-2.79)	<.001	1.18 (0.95-1.47)	.13
Patient experience					
≥National average	4195 (79.3)	1 [Reference]	NA	1 [Reference]	NA
<National average	1093 (20.7)	1.78 (1.55-2.04)	<.001	1.63 (1.37-1.94)	<.001
Hospital type					
Acute care	3255 (61.6)	1 [Reference]	NA	1 [Reference]	NA
Psychiatric	582 (11.0)	0.36 (0.30-0.44)	<.001	0.43 (0.33-0.54)	<.001
Critical access	1355 (25.6)	0.75 (0.65-0.85)	<.001	0.98 (0.81-1.17)	.79
Children’s	96 (1.8)	0.57 (0.38-0.86)	<.001	0.69 (0.45-1.06)	.09
Hospital ownership					
Local government	1170 (22.1)	1 [Reference]	NA	1 [Reference]	NA
Federal government	59 (1.1)	0.67 (0.40-1.14)	.14	0.69 (0.40-1.18)	.18
Private nonprofit	2624 (49.6)	1.16 (1.00-1.34)	.05	0.97 (0.84-1.13)	.73
Religious	327 (6.2)	0.74 (0.58-0.95)	.02	0.56 (0.43-0.73)	<.001
Private for-profit	1108 (21.0)	1.30 (1.09-1.55)	<.001	1.32 (1.09-1.60)	<.001

Abbreviations: NA, not applicable; OR, odds ratio.

In univariable and multivariable regression analyses, below average patient experience (vs at or above average patient experience) and private nonprofit ownership (vs local government ownership) were associated with greater compliance (Table). In contrast, psychiatric hospitals (vs acute care hospitals) and religious ownership (vs local government ownership) were associated with lesser compliance (Table).

To assess chargemaster usability, 2 nonmedical reviewers assessed 25 shoppable items in the chargemasters of the 100 largest hospitals by bed number. Across all items, 330 prices (13.2%) were identified by both reviewers, and prices were more frequently identified for medications and laboratory tests than for imaging. Agreement in abstracted prices ranged from 0% to 89% (Figure). Among 22 hospitals with chargemasters including *Current Procedural Terminology* (*CPT*) codes, 370 of 551 prices (67.2%) across all 25 items were identified by *CPT* codes. However, only 21 of 57 prices (36.8%) abstracted by either nonmedical reviewer was concordant with pricing identified using *CPT* codes.

**Figure.**
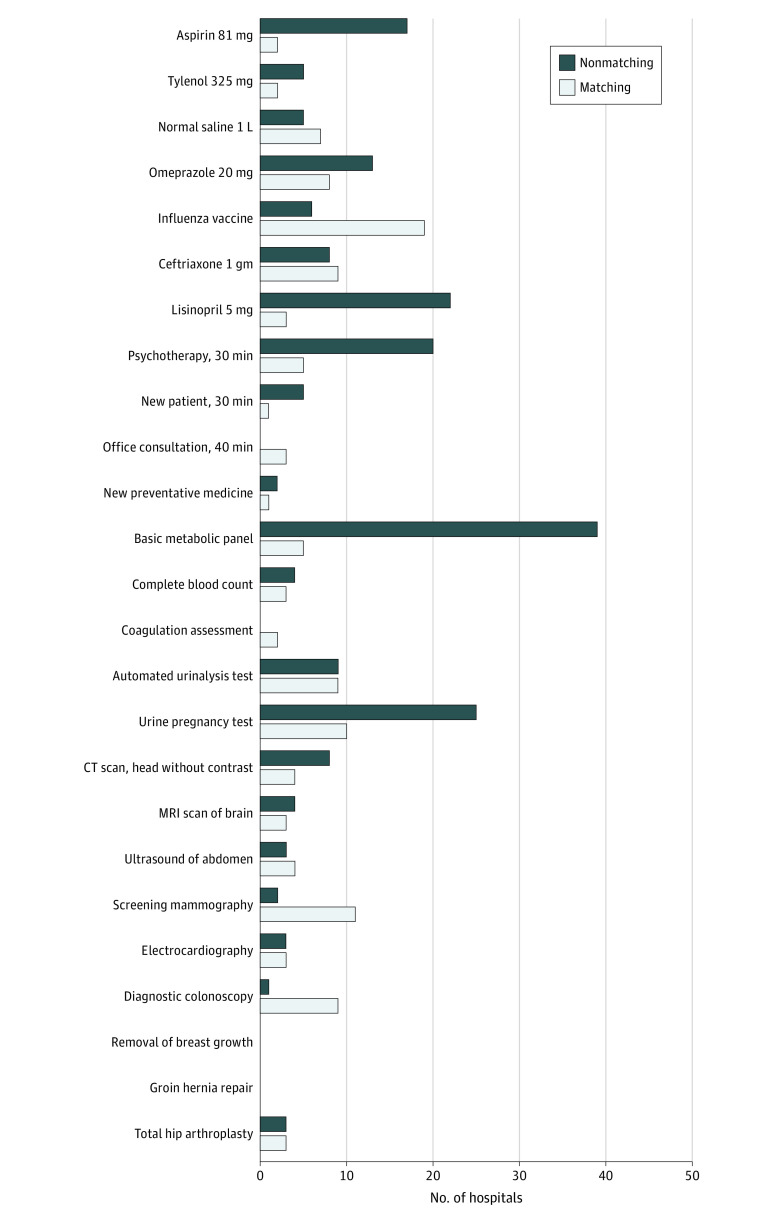
Chargemaster Price Identification and Variation for Shoppable Items Across the 100 Largest US Hospitals Number of hospitals with matching and nonmatching prices for 25 shoppable items between 2 nonmedical reviewers. CT indicates computed tomography; MRI, magnetic resonance imaging.

## Discussion

Our study found that most US hospitals remained noncompliant with the CMS-1694-F ruling, and compliance was associated with patient ratings, hospital type, and ownership. Even when publicly accessible, chargemasters were frequently buried within websites and difficult to use accurately. This work calls into question the effectiveness of CMS rulings to promote price transparency and highlights the challenges of creating effective price transparency tools for consumers. Additional data, including negotiated rates mandated in the final rule on price transparency (CMS-1717-F2), may improve the interpretability of hospital prices, but that rule does not address improving access and usability of hospital pricing data.^6^ One limitation of this study is that hospital pricing data may not reflect actual charges or costs.

## References

[1] Papanicolas I, Woskie LR, Jha AK. Health care spending in the United States and other high-income countries. JAMA. 2018;319(10):1024-1039. doi:10.1001/jama.2018.1150 29536101

[2] Emanuel E, Tanden N, Altman S, . A systemic approach to containing health care spending. N Engl J Med. 2012;367(10):949-954. doi:10.1056/NEJMsb1205901 22852883

[3] Sinaiko AD. What is the value of market-wide health care price transparency? JAMA. 2019. doi:10.1001/jama.2019.11578 31486830

[4] Wu SJ, Sylwestrzak G, Shah C, DeVries A. Price transparency for MRIs increased use of less costly providers and triggered provider competition. Health Aff (Millwood). 2014;33(8):1391-1398. doi:10.1377/hlthaff.2014.0168 25092841

[5] Centers for Medicare & Medicaid Services. 10 Steps to making public standard charges for shoppable services. Accessed April 6, 2021. https://www.cms.gov/files/document/steps-making-public-standard-charges-shoppable-services.pdf

[6] Desai S, Hatfield LA, Hicks AL, Chernew ME, Mehrotra A. Association between availability of a price transparency tool and outpatient spending. JAMA. 2016;315(17):1874-1881. doi:10.1001/jama.2016.4288 27139060

